# Sporadic and recurrent non-suicidal self-injury before age 14 and incident onset of psychiatric disorders by 17 years: prospective cohort study

**DOI:** 10.1192/bjp.2017.45

**Published:** 2018-03-08

**Authors:** Paul O. Wilkinson, Tianyou Qiu, Sharon Neufeld, Peter B. Jones, Ian M. Goodyer

**Affiliations:** 1 University of Cambridge and Cambridgeshire and Peterborough NHS Foundation Trust, Cambridge, UK; 2 University of Cambridge, Cambridge, UK and University of British Columbia, Vancouver, Canada; 3 University of Cambridge, Cambridge, UK; 4 University of Cambridge and Cambridgeshire and Peterborough NHS Foundation Trust, Cambridge, UK

## Abstract

**Background:**

Non-suicidal self-injury (NSSI) is highly prevalent in adolescents and may be a behavioural marker for emergent mental illnesses.

**Aims:**

To determine whether sporadic or recurrent NSSI up to the age of 14 years predicted increased risk of new onset of psychiatric disorder in the subsequent 3 years, independent of psychiatric symptoms and social risk factors.

**Method:**

In total, 945 individuals aged 14 years with no past/present history of mental illness completed a clinical interview and completed a questionnaire about NSSI at the ages of 14 and 17 years.

**Results:**

Recurrent NSSI at baseline predicted total disorders, depression and eating disorders. Sporadic baseline NSSI predicted new onset of anxiety disorders only.

**Conclusions:**

NSSI (especially recurrent NSSI) in the early-adolescent years is a behavioural marker of newly emerging mental illnesses. Professionals should treat both recurrent and sporadic NSSI as important risk factors, and prevention strategies could be targeted at this vulnerable group.

Prevalence of self-harm is high, varying from 8 to 25% in recent community surveys of young people.^1^^–^^3^ Reduction of self-harm is a key public health target in the UK and internationally.^4^ Although some self-harm is attempted suicide, there is increasing interest in the phenomenon of non-suicidal self-injury (NSSI) – harming the surface of one's body but without any intent to end life. Motivations include: to relieve distressing affect, self-punishment, to communicate distress to others, to fit in with peers.^5^ This delineation between suicidal and non-suicidal self-harm is controversial, with some considering the distinction from suicide attempts valid but others arguing that suicidal and non-suicidal self-harm are related behaviours and should not be separated given that intent is often not clear, and the fact that many people engage in both.^6^^–^^9^

If the behaviour of NSSI is discernible and is indeed ‘non-suicidal’, is it of itself clinically important given the large proportion of adolescents with NSSI but no history of mental illness?^10^^–^^12^ A recent community cohort study has demonstrated that NSSI by the age of 16 predicted depressive and anxiety disorders and substance use at age 18.^11^ NSSI has also been associated with future attempted and completed suicide, in all age groups.^13^^,^^14^ According to Joiner's interpersonal-psychological theory of suicidal behavior, NSSI may increase suicide risk through increased habituation to physical pain.^15^ Most adolescents who self-harm stop doing so by their twenties, so for many it is a temporary hazardous behaviour, confined to adolescence.^2^ Some adolescents try NSSI once, others engage in it hundreds of times. A recent international epidemiological study demonstrated that only 37% of adolescents who engage in NSSI do so more than four times in their life, suggesting it is a low-frequency sporadic behaviour in the majority.^1^ Repeated self-harm is associated with higher risk of completed suicide than single episodes.^16^ Whether repeated NSSI is a stronger marker for risk of subsequent mental illness than single-episode NSSI is not known.^17^ And whether single episodes of NSSI predict future mental illness is not known.

NSSI may be a relatively common and non-specific risk for subsequent mental illness but may itself be a correlated outcome of other established social-risk experiences. For example exposure to childhood adversity is known to increase the risk for both NSSI and mental illness, including in this sample of adolescents.^17^^,^^18^ In addition, more proximal environmental adversities, such as current peer group or family difficulties are also likely to increase risk of both mental illness and NSSI.^17^^,^^19^ To establish a valid correlation between NSSI and subsequent psychiatric disorder requires the known effects of childhood maltreatment and proximal environmental difficulties to have been taken into account. Some individuals with NSSI may be at conjoint risk for this hazardous behaviour and subsequent psychiatric caseness because of an underlying common general emotional/behavioural liability towards both. In this sample such a general latent distress trait has been reported^20^ and is associated with the emergence of all mental illnesses and NSSI over the 14- to 17-year age range (available from authors on request).^21^ Here, we report a prospective study that investigates the general hypothesis that NSSI by 14 years of age is a behavioural marker for incident onset mental illness by 17 years. More specifically we hypothesise that any such association is confined to those with recurrent episodes of NSSI. We reason that single-episode NSSI may be a sporadic event in the teen population at large of no marked prognostic significance. We test whether there is an independent association between NSSI and subsequent mental illness, or whether this is entirely confounded by being located at the higher end of a general latent distress trait and/or environmental adversity.

## Method

### Sample

In total, 1238 adolescents aged 14 years old were recruited from schools in Cambridgeshire and Suffolk, UK, in 2005–2007, for the Roots study. Recruitment was by invitation letters and (for 14/18 schools) presentations in school assemblies. Participants had follow-up interviews at the age of 17, from 2008 to 2010. Further details of sample recruitment and representativeness are found elsewhere.^22^

### Measures

NSSI was measured by a binary self-report question: ‘Have you ever tried to hurt yourself on purpose, without trying to kill yourself (for example burning, cutting, or scratching yourself)?’ as part of a new Drugs and Self Injury Questionnaire (DASI), a ten-item self-report measure assessing risk-taking behaviour. A further question established frequency of NSSI; participants were asked to mark one of four frequency bands, relating to maximum number of episodes of NSSI per year: never, once, 2–3, 4 or more. Groups were classified as two or more times per year (recurrent NSSI), maximum once per year (sporadic NSSI) and never NSSI, in keeping with our *a priori* hypothesis. Use of a single item to measure the discrete behaviour of self-harm is consistent with other published literature in the field.^11^ Reliability and validity of the DASI have been demonstrated using two methods: NSSI prevalence was nearly identical in two independent community cohort studies both at ages 14 (cohort effect odds ratio (OR) = 0.98) and 17 (cohort effect OR = 1.00), once differences in demographic, psychological and environmental variables were controlled for (details available from the authors on request). Second, the DASI item showed high convergent validity with the multi-item Self-Harm Inventory^23^ (SHI, point-biserial correlation between the SHI total and DASI self-ham question *r* = 0.66, *P*<0.0005) in a third sample (details available from the authors on request).

Presence or absence of DSM-IV psychiatric disorder^24^ was established by the Kiddie-Schedule for Affective Disorders and Schizophrenia-Present and Lifetime Version (K-SADS-PL) interviews at the ages of 14 and 17 years.^25^ All anxiety disorders were combined to one group because of sample size. An ‘any psychiatric diagnosis’ variable was scored as positive if participants scored ‘yes’ for any diagnosis (DSM-IV depressive disorders, anxiety disorders, eating disorders, behaviour disorders, substance use disorders, together with participants with respective threshold disorders defined as full psychosocial impairment, but one fewer symptom than needed for DSM-IV diagnosis). Participants with psychiatric diagnosis before/at the age of 14 were excluded to remove the potential for reverse causality (psychiatric illness → NSSI).

### Potential confounders

Depressive symptoms were measured by the Mood and Feelings Questionnaire^26^ and anxiety symptoms by the Revised Children's Manifest Anxiety Scale.^27^ From these items a bifactor analysis revealed an underlying ‘general distress factor’ shown to predict onsets of depressive, anxiety and behavioural disorders between 14 and 17 years in this cohort.^20^^,^^21^

Evaluation of the early family environment and exposure to adversities before the age of 5 years was measured by information obtained from one parent respondent (>90% biological mothers) using the Cambridge Early Experiences Interview.^18^ Latent class analysis was used to assign all participants to one of four groups: optimal family environment (63%) and three suboptimal ones labelled atypical (7%), discordant (24%) and hazardous family environments (6%). The latter three all implicate exposure to an adverse parenting experience. For the purposes of this analysis, the latter three groups were collapsed into one ‘childhood adversity’ group.

A parent/caregiver rated the general functioning subscale of the McMaster Family Assessment Device, to measure baseline family functioning.^28^ The Cambridge Friendships Questionnaire was used to measure peer relationships at baseline.^29^ This questionnaire contains eight items including both positive (for example ‘Can you confide in your friends?’) and negative (for example ‘Do people who aren't your friends laugh at you or tease you in a hurtful way?’) aspects of peer relationships.

A Classification of Residential Neighborhoods (ACORN) was used to capture the socioeconomic status of participants, derived via postcodes (www.caci.co.uk). ACORN categories were collapsed into three groups – low (hard-pressed and moderate means), middle (comfortably off) and high (urban prosperity and wealthy achiever) socioeconomic status.

### Statistical analysis

Logistic regressions were performed with presence/absence of psychiatric disorder as the outcome variable. As the purpose of the analysis was to test for predictors of onset of new disorder aged 14–17, participants with past or present disorder at 14 were excluded. Regressions compared groups defined by NSSI: sporadic *v.* never, recurrent *v.* never and recurrent *v.* sporadic. Three levels of hierarchical regressions were performed: the first model included NSSI, gender and socioeconomic status as covariates; the second model also included baseline psychiatric symptoms at age 14 (general distress); the third model also included early environmental adversity, baseline peer relationships and baseline family function. An effect size (equivalent to Cohen's *d*) was estimated for the NSSI-disorder association in model 3, using the method of Chinn.^30^ Analyses were conducted on Stata 14. The Roots study was approved by the Cambridgeshire two local research ethics committee.

## Results

In total, 1198/1238 (97%) adolescents had baseline data on psychiatric disorder and NSSI, with 12% (*n* = 144) reporting any lifetime NSSI. We excluded 252 (21%) because of past or current psychiatric disorder, of whom 76 (30%) had a history of NSSI. Of the remaining adolescents, 27 (2.9%) had recurrent NSSI (2–3 times per year: 14; at least four times per year: 13); 40 (4.2%) had sporadic NSSI; one reported NSSI but not frequency, and was excluded from further analysis. Of the final 945 participants, 443 (47%) were male and 842/911 (92%) with ethnicity information were White. There was data on diagnosis at follow-up for 848 (90%). Demographic information on the baseline sample and those with follow-up data is presented in Table 1. Those with and without follow-up data did not differ in terms of gender, ethnic group (white *v.* other), baseline general distress or socioeconomic status (all *P* > 0.5). However, those with recurrent NSSI were more likely to remain in the study than those with less frequent NSSI (likelihood ratio χ^2^(d.f.=2) = 7.7, *P* = 0.021).

**Table 1:** Baseline sample demographics for whole sample and those with follow-up data

	Baseline sample (*n* = 945)	Sample with follow-up data (*n* = 848)
Gender, *n* (%)		
Male	443 (47)	386 (46)
Female	502 (53)	462 (54)
Socioeconomic status, *n* (%)		
Wealthy achievers/urban prosperity	595 (63)	537 (63)
Comfortably off	225 (24)	204 (24)
Moderate means/hard-pressed	125 (13)	107 (13)
Ethnicity (baseline *n* = 911), *n* (%)		
White	842 (92)	763 (92)
Asian	15 (1.6)	13 (1.6)
Chinese	7 (0.8)	7 (0.9)
Black	5 (0.5)	3 (0.4)
Mixed/other	42 (4.6)	40 (4.8)
Baseline general distress (baseline *n* = 916), mean (s.d.)	−0.099 (0.612)	−0.106 (0.616)
Baseline NSSI (baseline *n* = 945), *n* (%)		
None	878 (93)	788 (93)
Sporadic	40 (4.2)	33 (3.9)
Recurrent	27 (2.9)	27 (3.2)

NSSI, non-suicidal self-injury.

**Fig. 1:**
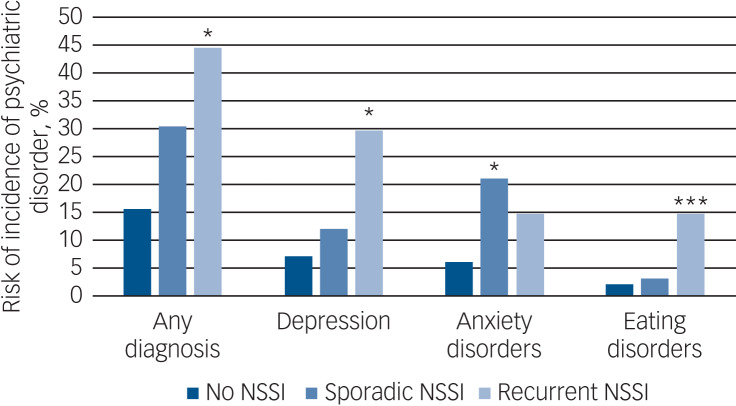
Unadjusted risk of incidence of psychiatric disorder aged 14–17 dependent on non-suicidal self-injury (NSSI) before the age of 14.

Table 2 shows the proportion of participants with a new psychiatric diagnosis in the follow-up period. In total, 17% of participants had a new onset of at least one disorder, with the most common disorders being major depression (8%) and anxiety disorders (7%). Table 3 and Fig. 1 show the associations between NSSI and future diagnoses. Results are not presented for disruptive behaviour disorders (conduct and oppositional disorders) and alcohol/substance use disorders because of the small numbers in the sample, (and only 1–2 of those with baseline NSSI having onset of a new disorder).

Adjusted analyses, group *v.* no NSSI: *P≤0.05, **P≤0.01, ***P≤0.001 (analyses adjusted for gender, socioeconomic status, baseline general distress, early environmental adversity, baseline peer relationships and baseline family function).

**Table 2:** New incidences of psychiatric diagnoses from ages 14 to 17

Diagnosis	Incidence (%)
Any	144/844 (17.1)
Major depression	68/848 (8.0)
Anxiety	60/848 (7.1)
Eating disorders	21/847 (2.5)
Behaviour disorders	11/842 (1.3)
Substance use disorder	10/845 (1.2)
Alcohol use disorder	18/847 (2.1)

**Table 3:** Associations between baseline non-suicidal self-injury and onset of psychiatric disorder

	Odds ratio (95% CI)			Estimated effect size for model 3
	Model 1^a^	Model 2^b^	Model 3^c^	
All diagnoses (incidence: 144/844, 17.1%)				
Sporadic *v.* no NSSI	2.06 (0.94–4.50)	1.62 (0.73–3.61)	1.78 (0.75–4.23)	0.32 (−0.15 to 0.80)
Recurrent *v.* no NSSI	4.32 (1.92–9.73)***	2.93 (1.26–6.84)*	3.20 (1.31–7.78)*	0.64 (0.15–1.13)*
Recurrent *v.* sporadic NSSI	2.01 (0.67–6.03)	2.09 (0.67–6.49)	1.84 (0.52–6.50)	0.34 (−0.36 to 1.03)
Depressive disorder (incidence: 68/848, 8.0%)				
Sporadic *v.* no NSSI	1.36 (0.45–4.08)	1.16 (0.38–3.54)	1.32 (0.41–4.21)	0.15 (−0.49 to 0.79)
Recurrent *v.* no NSSI	4.65 (1.90–11.38)***	3.35 (1.31–8.54)*	2.79 (1.02–7.66)*	0.57 (0.01–1.12)*
Recurrent *v.* sporadic NSSI	3.24 (0.82–12.75)	2.66 (0.64–11.13)	2.40 (0.50–11.46)	0.48 (−0.38 to 1.35)
Anxiety disorders (incidence: 60/848, 7.1%)				
Sporadic *v.* no NSSI	3.56 (1.44–8.78)**	2.99 (1.18–7.53)*	2.93 (1.05–8.16)*	0.59 (0.03–1.16)*
Recurrent *v.* no NSSI	2.45 (0.80–7.52)	1.85 (0.58–5.94)	2.07 (0.62–6.98)	0.40 (−0.26 to 1.07)
Recurrent *v.* sporadic NSSI	0.65 (0.16–2.57)	0.64 (0.15–2.65)	0.64 (0.14–3.00)	−0.25 (−1.09 to 0.61)
Eating disorders (incidence: 21/847, 2.5%)				
Sporadic *v.* no NSSI	1.09 (0.14–8.62)	1.00 (0.12–7.98)	Model did not fit	N/A
Recurrent *v.* no NSSI	6.22 (1.88–20.54)**	5.86 (1.66–20.72)**	9.96 (2.52–39.37)***	1.27 (0.51–2.03)
Recurrent *v.* sporadic NSSI	Model did not fit	Model did not fit	Model did not fit	N/A

NSSI, non-suicidal self-injury; N/A, not applicable.

a. Adjusted for gender, socioeconomic status (SES).

b. Adjusted for gender, SES, baseline general distress.

c. Adjusted for gender, SES, baseline general distress, early environmental adversity, baseline peer relationships, and baseline family function.

*P ≤ 0.05, **P ≤ 0.01, ***P ≤ 0.001.

Participants with recurrent NSSI had significantly greater risk of any new psychiatric diagnosis than those with no NSSI (OR = 4.32, 95% CI 1.92–9.73). This association was attenuated but remained statistically significant after controlling for baseline general distress (OR = 2.93, 95% CI 1.26–6.84). Controlling for environment had little effect on this risk (OR = 3.20, 95% CI 1.31–7.78). The risk for those with sporadic NSSI at baseline was intermediate between those with none and recurrent NSSI, but differences were not statistically significant. Risk for depression was significantly higher in those with recurrent *v.* no baseline NSSI (OR = 4.65, 95% CI 1.90–11.38). This remained significant after controlling for baseline general distress and environment (OR = 2.79, 95% CI 1.02–7.66, effect size = 0.57). Sporadic NSSI was not associated with risk of depression (OR = 1.32, 95% CI 0.41–4.21, effect size = 0.15). Recurrent NSSI was associated with greater risk for onset of eating disorders than no NSSI, even after controlling for symptoms and environment (OR = 9.96, 95% CI 2.52–39.37).

A different pattern was seen for anxiety disorders. Risk was slightly (but non-significantly) greater in the sporadic compared with the recurrent NSSI group. Risk was significantly greater for those with sporadic *v.* no NSSI, even after controlling for symptoms and environment (OR = 2.93, 95% CI 1.05–8.16, effect size = 0.59), but not for those with recurrent NSSI (OR = 2.07, 95% CI 0.62–6.98, effect size = 0.40). It is important to state here that confidence intervals are in some cases, especially for individual disorders, quite wide, so those results need to be interpreted with caution.

## Discussion

Our paper set out to examine whether NSSI before the age of 14 predicted incidence of psychiatric illness between the ages of 14 and 17; to examine if this is only true for recurrent NSSI; and to test whether any association could be the result of confounding by common environmental risk factors or common underlying latent traits for psychological distress.

We replicated the findings of the UK Avon Longitudinal Study of Parents and Children (ALSPAC) study that adolescent NSSI does predict onset of psychiatric disorder in later adolescence.^11^ As with ALSPAC, this was not specific to any disorder, and rates were increased across depressive, anxiety and eating disorders. This finding in two independent prospective population-based cohort studies demonstrates that services should take NSSI seriously, as it is not just suicide attempts that indicate increased risk of poor outcomes. Our age range for onsets was younger (14–17) than ALSPAC (16–18), and we have thus demonstrated that pre-/early-adolescent NSSI (not just later NSSI) is also a strong risk marker.

As predicted, adolescents with recurrent NSSI were at greatest risk for onset of future psychiatric disorder. An exception to this was our finding that sporadic NSSI (defined as no more than once per year) led to significantly greater risk of onset of anxiety disorders. The lack of significant association between recurrent NSSI and anxiety disorders may be a type 2 error, given that the effect size was close to the ‘moderate’ threshold of 0.5. However, the lack of significant association between sporadic NSSI and depression is unlikely to be a meaningful type 2 error, given the low effect size (0.15).

Our study is the first to compare outcomes of sporadic (once per year) against more recurrent NSSI. We found that even NSSI engaged in only once per year carries greatly increased risk of onset of anxiety disorders. Thus, professionals need to take adolescents seriously when they present even with a single episode of NSSI. They are at greatly increased risk of onset of mental illness, and appropriate and effective help for them at this stage may mitigate risk.

### Potential explanations for the association between NSSI and future mental illness

A possible explanation for the NSSI–mental illness association is that there is a common latent distress trait liability on which both NSSI and mental illness are located. Therefore, adolescents with higher levels of psychological distress may be more likely to engage in NSSI and meet criteria for a full psychiatric disorder. Controlling for general distress at the age of 14 did lead to some attenuation of our NSSI–illness associations, particularly for depression. But this attenuation was only partial. However, participants may have had a subthreshold episode of symptoms (which led to NSSI) before the age 14, but recovered by the time of the assessment at age 14. They may have then developed an episode of DSM-IV disorder between 14 and 17. As we did not ask about symptoms at their worst pre-14, we cannot rule this out. Thus, it is possible that the common latent distress vulnerability is present, but symptoms are fluctuating.

It is also possible that common environmental risk factors lead to this shared risk for NSSI and mental illness. We measured several potential common environmental risk factors: early family adversity, family functioning at 14 as rated by a parent (and hence not confounded by adolescent symptoms) and friendship functioning at 14. Controlling for these aspects of social environment had little effect on the strength of our NSSI–illness associations, suggesting this is not an explanation.

We think chance and bias are unlikely explanations given that these findings have been found in two independent prospective cohort studies, in both cases with a low probability of type 1 errors. This leaves two possible explanations: residual confounding and causality.

#### Residual confounding

An observational study cannot measure all possible confounders. Although we measured current peer and family relationships and early adversity, these measures are likely not to capture all elements of these environments and there was no measure of the learning environment. However, we think it unlikely that unmeasured environment could account for the large proportion of the influence on disorder unaccounted for. It is also possible that biological factors (in particular genetic) may have been the common risk factors for NSSI and later disorder. Although this may have had some effect, it is unlikely to be anything like the full explanation: twin and adoption studies have demonstrated that only a small proportion of the risks for NSSI and adolescent diagnosis of depression are genetic.^31^^,^^32^

#### Causality

If results are not the result of confounding, there is the possibility that early-adolescent NSSI may directly increase the risk for later psychiatric illness. Although NSSI can lead to short-term relief of negative affect, it also leads to a range of negative social and psychological consequences, including feelings of shame or guilt, teasing and rejection by peers, or negative sanctions from teachers and parents.^33^ Indeed, NSSI at age 12 has been demonstrated to be associated with later harsh punishment by parents.^34^ Such negative social consequences can lead to a deteriorating cycle of impaired social relationships and negative emotions, culminating in mental illness. Further longitudinal research is needed that delineates such negative consequences of NSSI and tests whether it mediates the associations between NSSI and later disorder.

### Strengths and limitations

The strengths of this study include the prospective measurement at multiple time points, and the 3-year follow-up, with little attrition. We excluded participants with mental illness (and those just below diagnosis thresholds) before the age of 14, thus reducing reverse causality (mental illness causing NSSI as well as later mental illness). We had a wide range of measures of the environment, which we were able to investigate as potential confounders for the NSSI–illness association.

As stated above, our main limitation is that we only measured distress at age 14, not at the worst point before then. Therefore, high levels of distress at the time of NSSI before baseline were not captured, and may have confounded results. Our sample had relatively high socioeconomic status compared with the general population, so results may not generalise to those of lower SES. Of note, self-harm and mental illness are associated with lower socioeconomic status and so rates of these may have been lower than in the general population. Our moderate sample size meant that we had limited power to delineate the effects of sporadic and recurrent NSSI, particularly for specific disorders; we did not find any significant differences between the effects of recurrent and sporadic NSSI. We are therefore unable to conclude that recurrent NSSI has a stronger effect on disorder risk than sporadic NSSI – just that recurrent NSSI is significantly associated with disorder risk. We did not collect data on suicide attempts, and so cannot compare outcomes of those with suicidal and non-suicidal self-harm. We also did not collect data on self-harm specifically between the ages 14 and 17, so we cannot provide data on whether NSSI predicted later self-harm.

In summary, this study demonstrates that NSSI, both recurrent and sporadic NSSI, is a strong marker for greatly increased risk of mental illness between the ages of 14 and 17, especially anxiety and depressive disorders. Professionals should therefore treat cases of NSSI seriously, and consider offering treatment to mitigate risks of future deterioration. Such an association is not likely to be solely because of the effects of common risk factors. Further research is needed to elucidate the mechanisms.

## References

[1] Plener PL, Libal G, Keller F, Fegert JM, Muehlenkamp JJ. An international comparison of adolescent non-suicidal self-injury (NSSI) and suicide attempts: Germany and the USA. Psychol Med 2009; 39: 1549–58. 19171079 10.1017/S0033291708005114

[2] Moran P, Coffey C, Romaniuk H, Olsson C, Borschmann R, Carlin JB, The natural history of self-harm from adolescence to young adulthood: a population-based cohort study. Lancet 2012; 379: 236–43. 22100201 10.1016/S0140-6736(11)61141-0

[3] Swannell SV, Martin GE, Page A, Hasking P, St John NJ. Prevalence of nonsuicidal self-injury in nonclinical samples: systematic review, meta-analysis and meta-regression. Suicide Life Threat Behav 2014; 44: 273–303. 24422986 10.1111/sltb.12070

[4] Department of Health. *Improving Outcomes and Supporting Transparency. Part 1A: A Public Health Outcomes Framework for England, 2013–2016* Department of Health, 2012 (https://www.gov.uk/government/uploads/system/uploads/attachment_data/file/263658/2901502_PHOF_Improving_Outcomes_PT1A_v1_1.pdf).

[5] Briere J, Gil E. Self-mutilation in clinical and general population samples: prevalence, correlates, and functions. Am J Orthopsychiatry 1998; 68: 609–20. 9809120 10.1037/h0080369

[6] Nock MK. Self-injury. Annu Rev Clin Psychol 2010; 6: 339–63. 20192787 10.1146/annurev.clinpsy.121208.131258

[7] Muehlenkamp JJ, Kerr PL. Untangling a complex web: how non-suicidal self-injury and suicide attempts differ. Prev Res 2010; 17: 8–10.20835367

[8] Victor SE, Klonsky ED. Correlates of suicide attempts among self-injurers: a meta-analysis. Clin Psychol Rev 2014; 34: 282–97. 24742496 10.1016/j.cpr.2014.03.005

[9] Kapur N, Cooper J, O'Connor RC, Hawton K. Non-suicidal self-injury v. attempted suicide: new diagnosis or false dichotomy? Br J Psychiatry 2013; 202: 326–8. 23637107 10.1192/bjp.bp.112.116111

[10] Nock MK, Joiner TE Jr, Gordon KH, Lloyd-Richardson E, Prinstein MJ. Non-suicidal self-injury among adolescents: diagnostic correlates and relation to suicide attempts. Psychiatry Res 2006; 144: 65–72. 16887199 10.1016/j.psychres.2006.05.010

[11] Mars B, Heron J, Crane C, Hawton K, Lewis G, Macleod J, Clinical and social outcomes of adolescent self harm: population based birth cohort study. BMJ 2014; 349: g5954. 25335825 10.1136/bmj.g5954 PMC4205277

[12] Hintikka J, Tolmunen T, Rissanen ML, Honkalampi K, Kylma J, Laukkanen E. Mental disorders in self-cutting adolescents. J Adolesc Health 2009; 44: 464–7. 19380094 10.1016/j.jadohealth.2008.10.003

[13] Cooper J, Kapur N, Webb R, Lawlor M, Guthrie E, Mackway-Jones K, Suicide after deliberate self-harm: a 4-year cohort study. Am J Psychiatry 2005; 162: 297–303. 15677594 10.1176/appi.ajp.162.2.297

[14] Wilkinson P, Kelvin R, Roberts C, Dubicka B, Goodyer I. Clinical and psychosocial predictors of suicide attempts and nonsuicidal self-injury in the adolescent depression antidepressants and psychotherapy trial (ADAPT). Am J Psychiatry 2011; 168: 495–501. 21285141 10.1176/appi.ajp.2010.10050718

[15] Ribeiro JD, Joiner TE. The interpersonal-psychological theory of suicidal behavior: current status and future directions. J Clin Psychol 2009; 65: 1291–9. 19827114 10.1002/jclp.20621

[16] Zahl DL, Hawton K. Repetition of deliberate self-harm and subsequent suicide risk: long-term follow-up study of 11,583 patients. Br J Psychiatry 2004; 185: 70–5. 15231558 10.1192/bjp.185.1.70

[17] Lowenstein LF. Youths who intentionally practise self-harm. Review of the recent research 2001–2004. Int J Adolesc Med Health 2005; 17: 225–30. 16231474 10.1515/ijamh.2005.17.3.225

[18] Dunn VJ, Abbott RA, Croudace TJ, Wilkinson P, Jones PB, Herbert J, Profiles of family-focused adverse experiences through childhood and early adolescence: the ROOTS project a community investigation of adolescent mental health. BMC Psychiatry 2011; 11: 109. 21736727 10.1186/1471-244X-11-109 PMC3199756

[19] van Harmelen AL, Gibson JL, St Clair MC, Owens M, Brodbeck J, Dunn V, Friendships and family support reduce subsequent depressive symptoms in at-risk adolescents. PLoS One 2016; 11: e0153715. 27144447 10.1371/journal.pone.0153715 PMC4856353

[20] Brodbeck J, Abbott RA, Goodyer IM, Croudace TJ. General and specific components of depression and anxiety in an adolescent population. BMC Psychiatry 2011; 11: 191. 22151586 10.1186/1471-244X-11-191 PMC3266209

[21] Brodbeck J, Goodyer IM, Abbott RA, Dunn VJ, St Clair MC, Owens M, General distress, hopelessness-suicidal ideation and worrying in adolescence: concurrent and predictive validity of a symptom-level bifactor model for clinical diagnoses. J Affect Disord 2014; 152–154: 299–305. 10.1016/j.jad.2013.09.029 PMC3878575 24238952

[22] Goodyer IM, Croudace T, Dunn V, Herbert J, Jones PB. Cohort profile: risk patterns and processes for psychopathology emerging during adolescence: the ROOTS project. Int J Epidemiology 2010; 39: 361–9. 10.1093/ije/dyp173 PMC2846441 19359258

[23] Sansone RA, Wiederman MW, Sansone LA. The self-harm inventory (SHI): development of a scale for identifying self-destructive behaviors and borderline personality disorder. J Clin Psychol 1998; 54: 973–83. 9811134 10.1002/(sici)1097-4679(199811)54:7<973::aid-jclp11>3.0.co;2-h

[24] American Psychiatric Association. Diagnostic and Statistical Manual of Mental Disorder (4th edn) (DSM-IV). APA, 1994.

[25] Kaufman J, Birmaher B, Brent D, Rao UM, Flynn C, Moreci P, Schedule for affective disorders and schizophrenia for school-age children-present and lifetime version (K-SADS-PL): initial reliability and validity data. J Am Acad Child Adolesc Psychiatry 1997; 36: 980–8. 9204677 10.1097/00004583-199707000-00021

[26] Costello EJ, Angold A. Scales to assess child and adolescent depression: checklists, screens and nets. J Am Acad Child Adolesc Psychiatry 1988; 27: 726–37. 3058677 10.1097/00004583-198811000-00011

[27] Reynolds CR, Richmond BO. What I think and feel: a revised measure of children's manifest anxiety. J Abnorm Child Psychol 1978; 6: 271–80. 670592 10.1007/BF00919131

[28] Epstein NB, Baldwin LM, Bishop DS. The McMaster family assessment device. J Marital Fam Ther 1983; 9: 171–80.

[29] Goodyer IM, Herbert J, Tamplin A, Secher SM, Pearson J. Short-term outcome of major depression: II. Life events, family dysfunction, and friendship difficulties as predictors of persistent disorder. J Am Acad Child Adolesc Psychiatry 1997; 36: 474–80. 9100421 10.1097/00004583-199704000-00009

[30] Chinn S. A simple method for converting an odds ratio to effect size for use in meta-analysis. Stat Med 2000; 19: 3127–31. 11113947 10.1002/1097-0258(20001130)19:22<3127::aid-sim784>3.0.co;2-m

[31] Rice F, Harold G, Thapar A. The genetic aetiology of childhood depression: a review. J Child Psychol Psychiatry 2002; 43: 65–79. 11848337 10.1111/1469-7610.00004

[32] Maciejewski DF, Creemers HE, Lynskey MT, Madden PA, Heath AC, Statham DJ, Overlapping genetic and environmental influences on nonsuicidal self-injury and suicidal ideation: different outcomes, same etiology? JAMA Psychiatry 2014; 71: 699–705. 24760386 10.1001/jamapsychiatry.2014.89 PMC4241464

[33] Wilkinson P. Non-suicidal self-injury. Eur Child Adolesc Psychiatry 2013; 22 (suppl 1): S75–9. 23202887 10.1007/s00787-012-0365-7

[34] Baetens I, Claes L, Onghena P, Grietens H, Van Leeuwen K, Pieters C, The effects of nonsuicidal self-injury on parenting behaviors: a longitudinal analyses of the perspective of the parent. Child Adolesc Psychiatry Ment Health 2015; 9: 24. 26157481 10.1186/s13034-015-0059-2 PMC4495844

